# Enhancing the Surface Sensitivity of Metallic Nanostructures Using Oblique-Angle-Induced Fano Resonances

**DOI:** 10.1038/srep33126

**Published:** 2016-09-09

**Authors:** Kuang-Li Lee, Chia-Chun Chang, Meng-Lin You, Ming-Yang Pan, Pei-Kuen Wei

**Affiliations:** 1 Research Center for Applied Sciences, Academia Sinica, 128, section 2, Academia Road, Nangkang, Taipei 11529, Taiwan; 2 Department of Optoelectronics, National Taiwan Ocean University, Keelung 20224, Taiwan; 3 Institute of Photonics Technologies, National Tsing Hua University, Hsinchu 30013, Taiwan; 4 Institute of Biophotonics, National Yang-Ming University, Taipei, Taiwan

## Abstract

Surface sensitivity is an important factor that determines the minimum amount of biomolecules detected by surface plasmon resonance (SPR) sensors. We propose the use of oblique-angle-induced Fano resonances caused by two-mode coupling or three-mode coupling between the localized SPR mode and long-range surface plasmon polariton modes to increase the surface sensitivities of silver capped nanoslits. The results indicate that the coupled resonance between the split SPR (−k_SPR_) and cavity modes (two-mode coupling) has a high wavelength sensitivity for small-angle incidence (2°) due to its short decay length. Additionally, three-mode coupling between the split SPR (−k_SPR_), substrate (+k_Sub_) and cavity modes has a high intensity sensitivity for large-angle incidence due to its short decay length, large resonance slope and enhanced transmission intensity. Compared to the wavelength measurement, the intensity measurement has a lower detectable (surface) concentration below 1 ng/ml (0.14 pg/mm^2^) and is reduced by at least 3 orders of magnitude. In addition, based on the calibration curve and current system noise, a theoretical detection limit of 2.73 pg/ml (0.38 fg/mm^2^) can be achieved. Such a surface concentration is close to that of prism-based SPR with phase measurement (0.1–0.2 fg/mm^2^ under a phase shift of 5 mdeg).

Surface plasmon resonance (SPR) sensing is a real-time and label-free detection technique that has been employed in many applications, including medical diagnostics, environmental monitoring and food safety^1^,^2^,^3^,^4^. Commercial sensing platforms utilize an optical prism to induce the propagation of surface plasmon polaritons in thin noble films and to enable real-time and label-free measurements of biomolecular binding affinity. In addition to the prism coupling method, periodic metal nanostructures offer a simpler method for SPR excitation. Metallic nanostructures such as nanohole and nanoslit arrays have been used for biosensing applications^5^,^6^,^7^,^8^,^9^,^10^,^11^,^12^,^13^,^14^,^15^. Compared to prism-based SPR sensors, periodic metallic nanostructures have a number of benefits, including their small detection volume, simple measurement and ease of multiple detections. They provide a feasible way to achieve chip-based, high-throughput and label-free detection for modern DNA and protein microarrays. To evaluate the quality of the biosensor, figure of merit (FOM) values are utilized. One of the FOM values, in wavelength units, is defined as *S*_*λ*_ (nm/RIU)/*fwhm* (nm), where *S*_*λ*_ is the linear regression slope for the refractive index dependence (bulk sensitivity), and *fwhm* is the resonance width of the plasmon resonance. The redshift of the resonance wavelength, caused by a small index change, Δ*n*, also induces a relative intensity change, Δ*I/I*, at a fixed wavelength near the resonance condition. An alternative FOM* value is proposed and defined as^16^,^17^


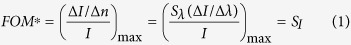


The FOM* value or intensity sensitivity (*S*_*I*_) is proportional to the bulk sensitivity and slope of the spectrum. However, both FOM values only consider bulk refractive index changes throughout the entire optical field and do not relate to a high surface sensitivity. High surface sensitivity is achieved when a greater proportion of the plasmonic field is occupied by an adsorbate layer^18^. When an analyte is attached to the surface of plasmonic sensors, the effective refractive index (*n*_*eff*_) observed on the surface plasmon can be expressed as follows:


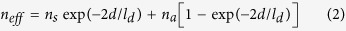


where *n*_*s*_ is the bulk solution refractive index, *n*_*a*_ is the adsorbate monolayer refractive index, *l*_*d*_ is the electromagnetic field decay length and *d* is the thickness of the adsorbate monolayer. For the surface plasmon wave propagating on a flat metal surface, the decay length *l*_*d*_ (where the amplitude drops to 1/e) is determined primarily by the resonance wavelength λ and can be express as follows^1^:


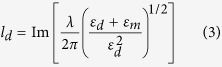


where *ε*_*m*_ and *ε*_*d*_ are the relative permittivities of the metal and the adjacent dielectric material. The shorter the resonance wavelength is, the shorter the decay length becomes. The wavelength shift (Δλ) caused by the monolayer can be expressed as


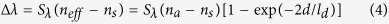


The spectral shift is related to the bulk wavelength sensitivity, the thickness of the adsorbate monolayer and the decay length. For biomolecular detection, *n*_*a*_ is similar, and the biomolecular thickness is the concern. Therefore, we defined the surface thickness sensitivity (*S*_*t*_) as Δλ/Δd, i.e., *S*_*t*_ = *2S*_*λ*_*(n*_*a*_ − *n*_*s*_*)/l*_*d*_. The quantity FoM_t_ is used instead of FoM* for the thickness measurement. FOM_t_ is defined as


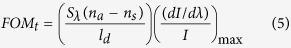


The surface sensing ability can be improved by increasing the bulk sensitivity, decreasing the decay length, increasing the refractive index difference between the adsorbate monolayer and surrounding environment or narrowing the bandwidth. For periodic metallic nanostructures, the bulk sensitivity can be increased with a longer period^14^,^19^. On the other hand, to reduce the bandwidth, a thermal-annealing method is used to produce high-quality metallic nanostructures. The annealed nanostructures have smoother metal surfaces and larger gold grains^19^,^20^,^21^,^22^,^23^, which reduce surface plasmon propagation loss and result in a sharp linewidth. Another approach to achieve a sharp spectral response is based on Fano resonances^24^,^25^,^26^. The Fano resonance exhibits a distinctly asymmetric shape, which arises from the spectral overlap between a broad resonance and a narrow discrete resonance^25^. The Fano resonances have been extensively studied in nanoparticles^5^, plasmonic nanostructures^26^,^27^ and metamaterials^28^. Additionally, the bandwidth of periodic nanostructures can be narrowed using angle-dependent transmitted spectra^27^,^29^. A narrower resonance can be obtained at a larger oblique angle. In addition, by tuning the incident angle, the angular momentum of the SPR mode at the metal/medium interface and the substrate mode at the substrate/metal interface can be matched. The coupling between both modes produces a sharp resonance peak, causes increased transmission intensity and enhances the FOM value^30^. Another study indicates that the SPR mode produces the highest intensity sensitivity at an angle smaller than the resonance angle^31^.

In this paper, we studied the optical properties of silver capped nanoslits with oblique-angle-induced Fano resonances for two-mode and three-mode coupling conditions. The sensing capabilities of multiple-Fano-coupling modes for wavelength and intensity interrogation were compared by measuring interactions between bovine serum albumin (BSA) and anti-BSA. We found that the wavelength shift caused by the anti-BSA adsorption for the coupled mode between the split SPR and cavity modes was improved by a factor of 1.9 when the incident angle is small. We verified that the improved surface sensitivity for the coupled mode was related to the decrease of the decay length. Additionally, large-angle incidence can induce three-mode coupling (BW-SPPs at air/metal, substrate/metal and gap plasmon), which has a more enhanced intensity. The limit of detection (LOD) for the intensity measurement was improved by a factor of 16. The improved LOD was attributed to the reduced decay length and enhanced transmission, which increased the spectral shift and resonance slope and simultaneously decreased the noise level. Two-mode coupling and three-mode coupling were suitable for wavelength and intensity measurements, respectively. The LOD, which was limited by the wavelength resolution (0.4 nm) of a miniature spectrometer, was 1 μg/ml for the wavelength interrogation. The intensity measurement had a much lower LOD, below 1 ng/ml (0.14 pg/mm^2^) and was reduced by at least 3 orders of magnitude. Such a surface concentration was better than that of the localized plasmon resonance in simple plasmonic nanostructures (1000 pg/mm^2^), bulky ATR sensors using an angular detection method (1 pg/mm^2^, with a resolution of 0.1 mdeg), and the quartz crystal microbalance (QCM) detection technique (20 pg/mm^2^, with a resolution of 0.1 Hz). In addition, based on the calibration curve and current system noise, a theoretical detection limit of 2.73 pg/ml (0.38 fg/mm^2^) can be achieved. Such a surface concentration is close to that of prism-based SPR with phase measurement (0.1–0.2 fg/mm^2^ under a phase shift of 5 mdeg).

## Results

### Optical properties of the silver capped nanoslits with oblique-angle incidence

**Figure 1:**
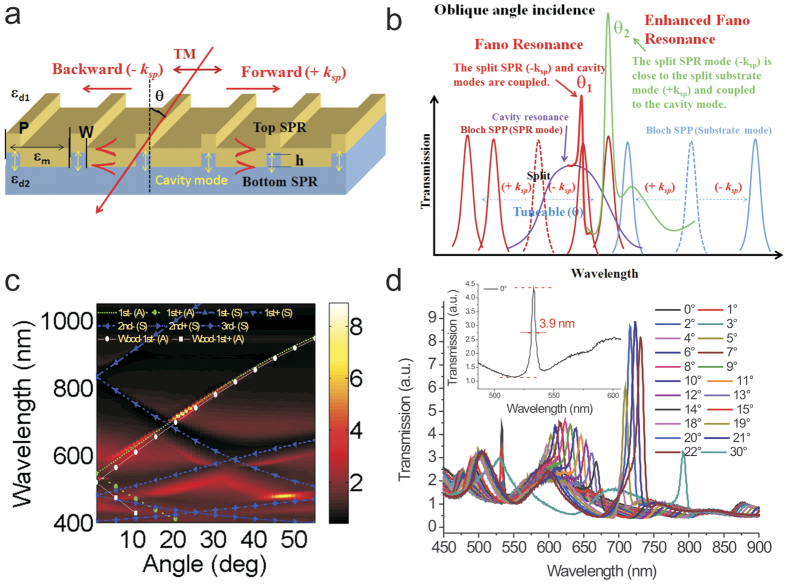
Optical properties of the silver capped nanoslits with oblique-angle incidence. (**a**) A schematic configuration depicts the geometrical parameters of the capped sliver nanoslits. (**b**) A schematic illustration demonstrates the Fano resonances (two- and three-mode coupling) in capped nanoslits under oblique-angle incidence. (**c**) The measured angular transmission diagram of 520-nm-period capped nanoslit arrays with an 80-nm-thick silver film in air for TM-polarized incident light. The green and blue dashed lines show the calculated resonance wavelengths (using equation (6)) for the SPR (the metal/air interface) and substrate modes (the metal/substrate interface), respectively. The white solid circle shows the calculated resonance wavelengths (using equation (7)) for first-order Wood’s anomaly at the metal/air interface. (**d**) The measured transmission spectra of 520-nm-period capped nanoslit arrays in air for different incident angles from 0° to 30°. The inset shows the bandwidth was 3.9 nm for normally incident TM-polarized light.

Figure 1a shows a schematic configuration depicting the geometrical parameters of the capped sliver nanoslits and the direction of the transverse magnetic (TM)-polarized incident light. Figure 1b shows a schematic illustration that demonstrates the Fano resonances in capped nanoslits under oblique-angle incidence. There are three resonance modes in the capped nanoslit arrays. One is the gap plasmon resonance (cavity mode) in the slit gaps, and the others are Bloch wave surface plasmon polaritons (BW-SPPs) on both sides of the periodic sliver surface (the silver/medium and silver/substrate interfaces). The cavity mode is coupled to the BW-SPP waves from the edges of the top and bottom interfaces. The light transmits through the nanoslits and capping layer, leading to a broadband transmission within the cavity spectrum. The resonance condition is estimated using a Fabry-Perot cavity^32^. The resonance wavelength is determined by the gap width and cavity length. The BW-SPP occurs on the periodic metallic surface when the Bragg condition is satisfied. The BW-SPP wave is scattered by the periodic grooves, resulting in narrowband transmission within the SPR spectrum. Under oblique-angle incidence, the Bragg condition for one-dimensional arrays can be described by ref. 1


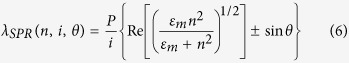


where *i* is the resonance order, *P* is the period of the nanostructure, *θ* is the incident angle, and *ε*_*m*_ and *n* are the dielectric constant of the metal and the environmental refractive index, respectively. The BW-SPP is split into the forward (+) and backward (−) propagating SPR modes. The resonance wavelength is controlled by the period and incident angle. In the case of capped nanoslit arrays with a 520-nm-period, 80-nm-gap and 80-nm-thick silver film, the cavity mode occurs at ~600 nm. The BW-SPP is at 530 nm when *θ* is zero. There is no spectral overlapping. For small-angle incidence (*θ*_*1*_), the backward-propagating SPR mode (a narrow discrete resonance) overlaps with the cavity mode (a broad resonance). This two-mode coupling generates an asymmetric Fano resonance^25^,^33^,^34^. By increasing the incident angle (*θ*_*2*_), the backward BW-SPP mode at the metal/air interface interacts with the cavity mode and the forward BW-SPP at the metal/substrate interface (the substrate mode). This three-mode coupling produces Fano resonance with enhanced transmission. Figure 1c shows the measured angular transmission diagram of 520-nm-period capped nanoslit arrays with an 80-nm-thick silver film in air for TM-polarized incident light. The green and blue dashed lines show the theoretical resonance wavelengths (calculated using equation (6)) for the SPR and substrate modes, respectively. The wavelength dependence permittivity of silver is obtained from Palik^35^. The experimental results were close to the calculated values. However, there is a larger deviation for very small angles. In this region, the slope is flat and angle-independent. We attribute the flat band to the domination of the cavity mode. Figure 1d shows the measured transmission spectra of 520-nm-period capped nanoslit arrays in air for different incident angles from 0° to 30°. For an incident angle of 0°, the resonance wavelengths of the cavity and SPR modes were approximately 600 and 534 nm, respectively. The inset shows that the bandwidth was 3.9 nm for normally incident TM-polarized light. As the incident angle increased from 0° to 15°, the backward-propagating SPR mode coupled to the cavity mode and an asymmetric Fano resonance was generated in the transmission spectra. When the incident angle increased to 21°, a sharp Fano resonance caused by three resonance modes was observed. Compared to the resonance mode at an incident angle of 0°, the coupled mode enhanced the transmission.

### Surface sensitivity tests for different incident angles with wavelength interrogation by measuring the interactions between BSA and anti-BSA

**Figure 2:**
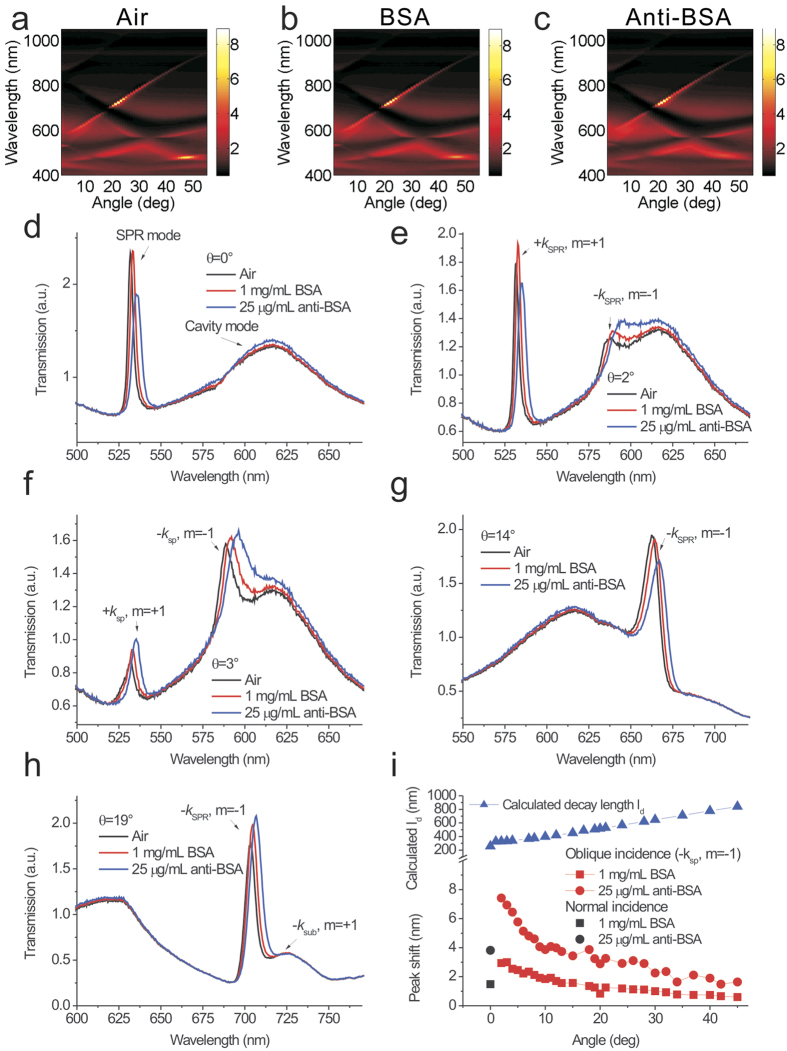
Surface sensitivity tests for different incident angles with wavelength interrogation by measuring the interactions between BSA and anti-BSA. The measured transmission diagram of the 520-nm-period silver capped nanoslits in (**a**), air, (**b**), 1 mg/mL BSA and (**c**), 25 μg/mL anti-BSA for different incident angles from 0° to 50°. The measured transmission spectra in air, 1 mg/mL BSA and 25 μg/mL anti-BSA for incident angles of (**d**), 0°, (**e**), 2°, (**f**), 3°, (**g**), 14° and (**h**), 19°. (**i**) Calculated decay length for a flat sliver surface and wavelength shift as a function of incident angle for BSA and anti-BSA adsorption.

We further studied the surface sensitivities of silver capped nanoslits under different incident angles from 0° to 55° by measuring the interactions between BSA and anti-BSA. Figure 2a–c show the measured transmission diagram of silver capped nanoslits in air, 1 mg/mL BSA and 25 μg/mL anti-BSA for different incident angles from 0° to 55°. Figure 2d–h show the measured transmission spectra in air, 1 mg/mL BSA and 25 μg/mL anti-BSA for incident angles of 0°, 2°, 3°, 14° and 19°. For an incident angle of 0°, the monolayer BSA and 150-kDa-sized anti-BSA resulted in red shifts of 1.49 and 3.82 nm, respectively. For an incident angle of 2°, the wavelength shifts for BSA and anti-BSA were increased to 2.94 and 7.42 nm, respectively. When the angle was increased to 19°, the BSA and anti-BSA wavelength shifts were reduced to 1.26 and 3.25 nm, respectively. The shifts were further decreased as the incident angle increased, as shown in Fig. 2i. The results indicate that the wavelength shift of the coupled resonance mode was improved by a factor of 1.94 for small-angle incidence (2°). For a spectral resolution of 0.4 nm, the detectable concentration of anti-BSA is 1.0 μg/mL for incident angles of 2°. Equation (4) demonstrates that once the analyte is chosen, the spectral shift is determined by the bulk sensitivity and decay length. For a surface plasmon wave propagating on a flat metal surface, the decay length is determined primarily by the resonance wavelength *λ*. The longer the resonance wavelength is, the shorter the decay length becomes. As θ increases, the resonance peak moves to a longer wavelength and the decay length increases, as indicated in Fig. 2h. This process results in a smaller wavelength shift because the overlap between the analyte and the SPR field decreases. Therefore, it was suggested that the wavelength shift will decrease as the incident angle increases. However, Fig. 2i shows that the wavelength shift first dramatically increased and then decreased as θ increased. The dramatic redshift occurred when the backward-propagating SPR mode coupled to the broadband cavity mode.

### Refractive index sensing capabilities of the silver capped nanoslits with oblique-angle incidence

**Figure 3:**
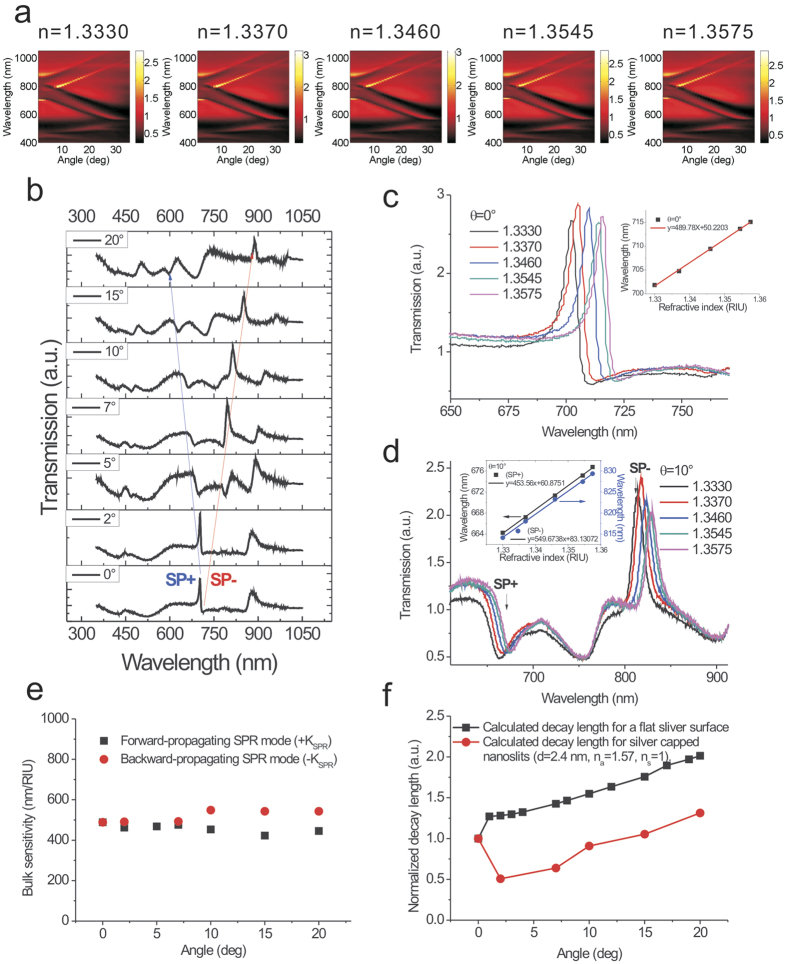
Refractive index sensing capabilities of the silver capped nanoslits with oblique-angle incidence. (**a**) A measured transmission diagram of the 520-nm-period silver capped nanoslits in different refractive index mixtures for different incident angles from 0° to 35°. (**b)** The measured transmission spectra in air for different incident angles from 0° to 20°. The measured transmission spectra in different refractive index mixtures for incident angles of (**c)**, 0° and (**d)**, 10°. The insets show the resonance wavelength against the refractive index for incident angles of 0° to 10°. (**e**) The bulk sensitivity versus the incident angle for different incident angles from 0° to 20°. (**f** ) The normalized decay lengths for a flat sliver surface and the capped nanoslits. The normalized decay lengths for the flat sliver surface were calculated using equation (3). The decay lengths for the capped nanoslits were calculated using equation (4). The measured bulk sensitivities and wavelength shifts caused by the adsorbate of BSA in Fig. 2i and the parameters *d* = 2.4 nm, *n*_*a*_ = 1.57 and *n*_*s*_ = 1 were utilized.

The spectral shift is dominated by the bulk sensitivity (*S*_λ_) and decay length (*l*_*d*_), as indicated in equation (4). To calculate the decay length, we first measured the bulk sensitivity by covering the capped nanoslit array with different refractive index media. Figure 3a shows the measured transmission diagram from 0° to 35°. Figure 3b shows the measured transmission spectra in air for different incident angles from 0° to 20°. As θ increases, the resonance peak splits into two resonances. Figure 3c,d show the measured transmission spectra in different refractive index mixtures for incident angles of 0° and 10°, respectively. The resonances were redshifted as the refractive index of the environment increased. There were linear correlations between the resonance wavelength and the refractive index of the environment for incident angles of 0° and 10° (see Fig. 3c inset and 3d inset). Figure 3e shows the bulk sensitivity versus the incident angle for different incident angles from 0° to 20°. The results show that when the incident angle changed from 0° to 20°, the bulk sensitivity decreased and increased within 10% for the forward- and backward-propagating coupled modes, respectively. According to equation (4), the decay lengths for different incident angles can be calculated with the measured bulk sensitivities, wavelength shifts and thicknesses of the biomolecules. Using the parameters *d* = 2.4 nm, *n*_*a*_ = 1.57 and *n*_*s*_ = 1, the normalized decay lengths of the capped nanoslits for different incident angles are shown in Fig. 3f. Compared to the normal incidence, the decay length first substantially decreased and then gradually increased as θ increased. Compared to the theoretical decay length for SPR on a flat sliver surface, the Fano resonance in the capped nanoslit had a much shorter decay length (see Fig. 3f). The decay lengths reduced by factors of 2.5 and 1.5 for incident angles of 2° and 20°, respectively.

### Calculated transmission spectra and resonance field distributions of the capped nanoslits using finite-difference time-domain (FDTD) calculations

**Figure 4:**
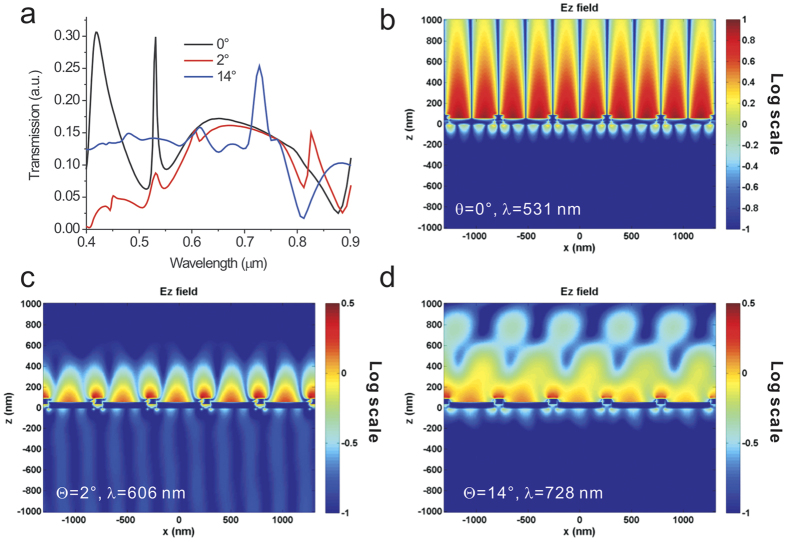
Calculated transmission spectra and resonance field distributions of the capped nanoslits using finite-difference time-domain (FDTD) calculations. (**a**) The calculated transmission spectra of the capped nanoslits for incident angles from 0° to 14° using the FDTD calculation method. There was an SPR resonance at a wavelength of 531 nm for normal incidence. It split, coupled to the cavity mode and redshifted to a wavelength of 606 nm when the incident angle increased to 2°. When the incident angle increased to 14°, the split SPR mode (−k_SPR_) is coupled to the cavity mode and close to the split substrate mode (+k_sub_). The resonance wavelength was at 727 nm. (**b**–**d**) The resonance field (E_z_) distributions for incident angles of 0° (**b**), 2° (**c**), and 14° (**d**).

We further utilized finite-difference time-domain (FDTD) calculations (FullWAVE 4.0, RSoft) to verify the decreased decay length for oblique incidence. Figure 4a shows the calculated transmission spectra of the capped nanoslits for incident angles from 0° to 14°. There were two distinct resonances in the spectrum for normal incidence. One was a sharp SPR resonance at a wavelength of 531 nm (the SPR mode). The other was a broadband resonance at 650 nm (the cavity mode). At an incident angle of 2°, the split SPR mode redshifted to the wavelength of 606 nm and coupled to the cavity mode. When the incident angle increased to 14°, the split SPR (−k_SPR_), substrate (+k_sub_) and cavity modes were coupled. An enhanced resonance peak at a wavelength of 727 nm was observed. Figure 4b–d show the resonance field (Ez) distributions for incident angles of 0°, 2° and 14°, respectively. The BW-SPP on the surface has a long evanescent tail for normal incidence. With a small increase of incident angle, the backward BW-SPP interacts with the cavity mode, and a Fano coupling mode occurs. The surface plasmon field is re-distributed. The cavity mode exhibits localized surface plasmon resonance in the nanoslits with a short decay length. It affects the field distribution of the Fano mode and results in a shorter decay length for the coupled resonance. Therefore, the improved wavelength sensitivity for the Fano-coupled mode is attributed to the decreased decay length. In addition to the BW-SPPs, Wood’s anomaly also occurs in periodic nanostructures. The resonance wavelength of Wood’s anomaly for one-dimensional arrays can be expressed as


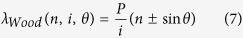


where *i* is the resonance order, *P* is the period of the nanostructure and *n* is the external refractive index. For metallic nanoparticle arrays, the particles are isolated. No BW-SPP features can be observed, and Wood’s anomaly (diffraction coupling) dominates the contribution^36^,^37^,^38^,^39^. In our case, very narrow slits were covered with a metallic film. The BW-SPP, Wood’s anomaly, and localized surface plasmon resonances may contribute to the spectrum. It has been reported that Wood’s anomalies are very narrow spectrally, and BW-SPPs, when present, tend to have a greater impact^34^. To verify the dominance of BW-SPPs, we added the theoretical curve of Wood’s anomaly to Fig. 1c. The measured dispersion curve for first-order resonances at the air/metal interface is close to the theoretically predicted BW-SPP mode using equation (6). In addition, the FDTD simulation results (Fig. 4b–d) also indicate that the near-field distributions are bound to the metal/dielectric interface and do not extend far from the metal surface, which indicates that the resonances are related to BW-SPPs. Therefore, in our case, BW-SPP features dominate the contribution and the observed resonances are caused by BW-SPPs.

### Surface sensitivity tests for different incident angles with intensity interrogation by measuring the interactions between BSA and anti-BSA

**Figure 5:**
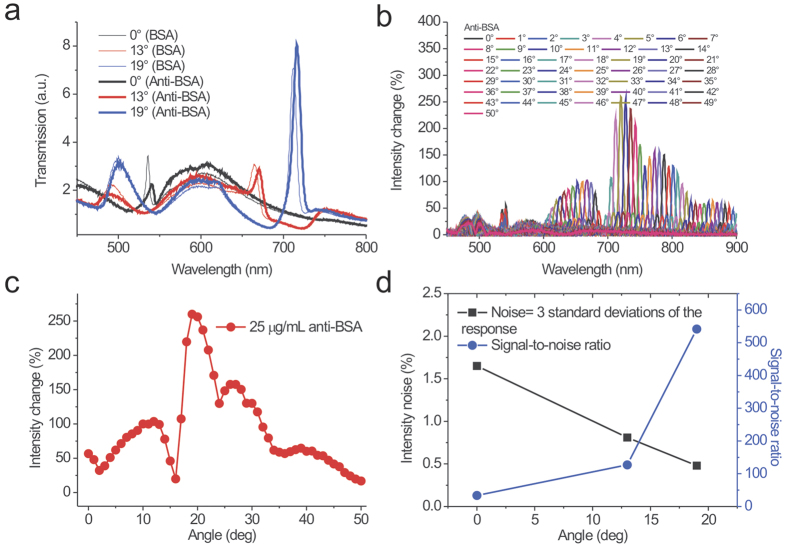
Surface sensitivity tests for different incident angles with intensity interrogation by measuring the interactions between BSA and anti-BSA. (**a**) The measured transmission spectra of 520-nm-period capped nanoslit arrays in air, BSA and anti-BSA adsorption conditions for incident angles of 0°, 13° and 19°. (**b**) The absolute spectral intensity changes caused by anti-BSA adsorption for different incident angles from 0° to 50°. (**c**) The maximum intensity changes as a function of incident angle for anti-BSA adsorption. (**d**) The intensity noise and signal-to-noise ratio (SNR) for incident angles of 0°, 13° and 19°. The intensity noise is defined as 3 standard deviations of the response.

We further compared the surface sensitivities for different incident angles using intensity interrogation. Figure 5a shows spectra for BSA and BSA-Anti-BSA at three different angles: 0°, 13° and 19°. Figure 5b shows the absolute spectral intensity changes 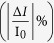 caused by anti-BSA adsorption for different incident angles from 0° to 50°. Figure 5c shows the maximum intensity changes as a function of the incident angle. The intensity changes were 56, 103 and 260% for incident angles of 0°, 13° and 19°, respectively. The maximum intensity change was at an angle of 19°, close to the three-mode coupling angle (21°). Compared to the normal incidence, the incident angle of 19° has enhanced the intensity change by a factor of 5. In addition, the measured intensity noises were 1.65%, 0.81% and 0.48% for incident angles of 0°, 13° and 19°, respectively, and the signal-to-noise ratios were 33, 127 and 542, respectively (see Fig. 5d). Obviously, the normal incident has a lower surface sensitivity. The signal-to-noise ratios were improved by factors of 3.8 and 16.4 for incident angles of 13° and 19°, respectively. From equation (5), the surface sensing ability is determined by the bulk sensitivity, the decay length and the resonance slope. For an incident angle of 19°, the backward BW-SPP at the metal/air interface interacted with the cavity mode and was close to the forward BW-SPP at the metal/substrate interface (see Fig. 1c). The split SPR mode (−k_SPR_) coupled to the cavity mode, which had a decreased decay length. In addition, the coupling between the split SPR (−k_SPR_) and substrate modes (+k_Sub_) enhanced peak transmission, which resulted in a larger resonance slope and reduced the noise. Therefore, the larger resonance slope, decreased decay length and lower noise enhanced the intensity detection for the incident angle of 19°. It was noted that although the wavelength sensitivity was high for the incident angle of 2°, the intensity sensitivity was low at this angle because of the small resonance slope. The above results indicate that the coupled resonance between the split SPR (−k_SPR_) and cavity modes (two-mode coupling) had a higher wavelength sensitivity for small-angle incidence (2°) due to its shorter decay length. However, three-mode coupling between the split SPR (−k_SPR_), substrate (+k_Sub_) and cavity modes at large-angle incidence had a higher intensity sensitivity due to the reduced decay length, larger resonance slope and enhanced transmission intensity. It was noted that for the three-mode coupling, the transmission is primarily enhanced by the resonance coupling between the substrate BW-SPP and surface BW-SPP modes. An oblique angle will split the surface BW-SPP (−k_SPR_) and substrate BW-SPP (+k_Sub_). At a certain angle (~21° in our case), the SP propagation constants on both sides of the nanoslits are matched and result in enhanced transmission. Such transmission enhancement has also been observed in nanohole arrays when the SP resonance energies on both sides of the metal film are matched^40^,^41^,^42^. For three-mode coupling, the resonance coupling of both BW-SPP modes is further coupled with the cavity mode and forms the Fano coupling mode. The surface plasmon field is re-distributed. The cavity mode exhibits localized surface plasmon resonance in the nanoslits with a short decay length, which affects the field distribution of the plasmon mode and results in a shorter decay length for the coupled resonance, as shown in Fig. 4d. Therefore, the transmission intensity and surface sensitivity are greatly enhanced by using three-mode coupling.

### Comparison of surface sensitivities with wavelength and intensity interrogation using 500-nm-period silver capped nanoslits

**Figure 6:**
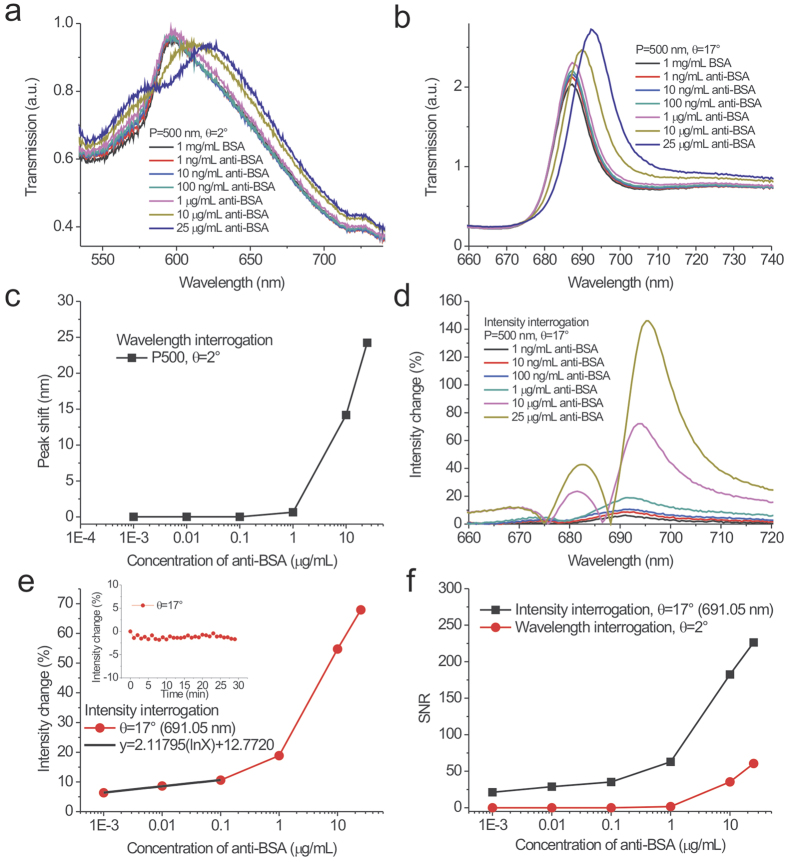
Comparison of surface sensitivities with wavelength and intensity interrogation using 500-nm-period silver capped nanoslits. The measured transmission spectra in 1 mg/mL BSA and different concentrations of anti-BSA solutions from 1 ng/mL to 25 mg/mL for incident angles of (**a**), 2° and (**b**), 17° using 500-nm-period capped nanoslits. (**c**) The peak wavelength shift caused by different concentrations of anti-BSA solutions for the incident angle of 2°. The peak wavelength of the BSA solution was set as a reference. (**d**) The absolute spectral intensity changes caused by different concentrations of anti-BSA solutions for the incident angle of 17°. The transmission spectrum of the BSA solution was set as a reference. (**e**) The intensity change as a function of the logarithm of the concentration of the anti-BSA solution at a wavelength of 691 nm. The inset shows the intensity change at a wavelength of 691 nm as a function of time. (**f**) The signal-to-noise ratios (SNR) as a function of the concentrations of the anti-BSA solution with wavelength and intensity interrogation at incident angles of 2° and 17°, respectively.

We further studied the LOD of 500-nm-period capped nanoslits by detecting different concentrations of anti-BSA solutions with wavelength and intensity interrogation. Figure 6a,b show the measured transmission spectra in 1 mg/mL BSA and different concentrations of anti-BSA solutions from 1 ng/mL to 25 μg/mL for incident angles of 2° and 17°, respectively. The resonance peaks were redshifted as the concentrations increased and the transmitted intensities changed. We analyzed the spectra and set the transmission spectra of the BSA solutions as references. Figure 6c shows the peak wavelength shifts caused by different concentrations of anti-BSA solutions for the incident angle of 2°. The peak wavelength shifts were 0, 0, 0, 0.64, 14.1 and 24.2 nm for concentrations of 0.001, 0.01, 0.1, 1, 10 and 25 μg/ml, respectively. Obviously, concentrations of less than 0.1 μg/ml produced no response. The detectable concentration, which was limited by the wavelength resolution (0.4 nm) of the spectrometer, was 1 μg/ml with wavelength interrogation. Figure 6d shows the absolute spectral intensity changes caused by different concentrations of anti-BSA solutions for the incident angle of 17°. Obviously, even for a concentration of 1 ng/ml, the intensities had small changes at a wavelength of 691 nm. Figure 6e shows the intensity change as a function of the logarithm of the concentrations at a wavelength of 691 nm. The intensity change increased and then gradually saturated as the concentration increased. The responses were 6.3, 8.6, 10.5, 18.8, 54.7, 67.9% for 0.001, 0.01, 0.1, 1, 10, 25 μg/ml, respectively. There was a linear correlation between the response and the concentration when the concentration was less than 0.1 μg/ml. The calibration curve was described by y = 2.11795(ln(x)) + 12.7720, R^2^ = 0.99604. In addition, the measured intensity noise, extracted from the inset in Fig. 6c, was 0.33% (one standard deviation of the response). Therefore, the signal-to-noise ratios ranged from 21 to 226 for different concentrations. They were higher than that with the wavelength measurement, as shown in Fig. 6f. Compared to the wavelength measurement, the intensity measurement had a lower LOD (below 1 ng/ml). It decreased by at least 3 orders of magnitude. It has been reported that the surface concentration can be determined by the thickness, i.e., the surface concentration (μg/cm^2^) ≈ 0.12 × t (nm)^43^. For 10 μg/ml anti-BSA adsorption, the estimated surface concentration is 0.14 μg/cm^2^ for 1.2 nm thickness^44^. When the measured concentration was 1 ng/ml and the detectable surface concentration was below 0.14 pg/mm^2^. Such a surface concentration was better than that of the localized plasmon resonance in simple plasmonic nanostructures (1000 pg/mm^2^)^5^, the bulky ATR sensors using an angular detection method (1 pg/mm^2^ with a resolution of 0.1 mdeg)^45^,^46^,^47^, and the QCM detection technique (20 pg/mm^2^ with a resolution of 0.1 Hz). In addition, based on the calibration curve and current system noise (0.99%, 3 standard deviations of the response), the LOD of the surface concentration (detectable concentration) of anti-BSA can be obtained by a linear regression equation. This yields a theoretical detection limit of 2.73 pg/ml (0.38 fg/mm^2^). This detection limit was similar to that of plasmonic metamaterials with topological darkness (0.1 fg/mm^2^ with a phase noise of 5 mdeg)^48^ and graphene-protected copper plasmonics (0.2 fg/mm^2^ with a phase shift of 5 mdeg)^49^.

## Discussion

We studied the optical properties of the silver capped nanoslits with oblique incident angles and compared the sensing capabilities of the structures by detecting different concentrations of anti-BSA solutions via wavelength and intensity analysis. For normal light incidence, there was a sharp resonance peak in the transmission spectrum. The resonance bandwidth was 3.9 nm. Under oblique-angle incidence, the coupling of the cavity mode to the split Bloch wave surface plasmon polariton mode generated an asymmetric Fano resonance. The wavelength shift caused by the anti-BSA adsorption for the coupled mode was improved by a factor of 1.9 when the incident angle was increased to 2°. We verified that the improved surface sensitivity for the coupled mode (two-mode coupling) was related to the decreased decay length. For larger-angle incidence, the mode coupling (three-mode coupling) between the split SPR (−k_SPR_), substrate (+k_Sub_) and cavity modes enhanced the transmission of the asymmetric Fano resonance. Compared to the SPR mode, the larger-angle induced resonance mode had a higher signal-to-noise ratio using the intensity measurement. It was improved by a factor of 16. The improved surface sensitivity was attributed to the enhanced transmission, which increased the resonance slope and decreased the noise level at the same time. In the capped nanoslit arrays, two-mode and three-mode couplings were suitable for the wavelength and intensity measurements, respectively. However, the detectable concentration was 1 μg/ml for the wavelength interrogation for a common spectrometer, while the intensity measurement for three-mode coupling had a lower detectable (surface) concentration below 1 ng/ml (0.14 pg/mm^2^). It was reduced by at least 3 orders of magnitude compared to two-mode coupling using wavelength interrogation. In addition, based on the calibration curve and current system noise, it yielded a theoretical detection limit of 2.73 pg/ml (0.38 fg/mm^2^). For prism-based SPR, the structure was simple (a gold film on a prism) and the fabrication cost was low. An optical setup was necessary to precisely control the angle or phase resolution. By measuring the phase change, the limit of detection can be further improved. However, the proposed method utilized nanostructures and measured the intensity change at a fixed angle. The optical setup was simple, and a precise angle control system was not needed. Three-mode coupling can improve the surface sensitivity, which is close to that of prism-based SPR with phase measurement. However, advanced nanotechnology is required to fabricate these structures, and the fabrication cost is higher. In this study, we show the improved surface sensitivity of the capped nanoslit array. The idea of using split long-range surface plasmon polariton (SPP) modes (angle-dependent) coupled with localized SPR mode (angle-independent) to enhance surface sensitivity can also be applied to other periodic metallic nanostructures.

## Methods

### Fabrication of metallic nanostructures

**Figure 7:**
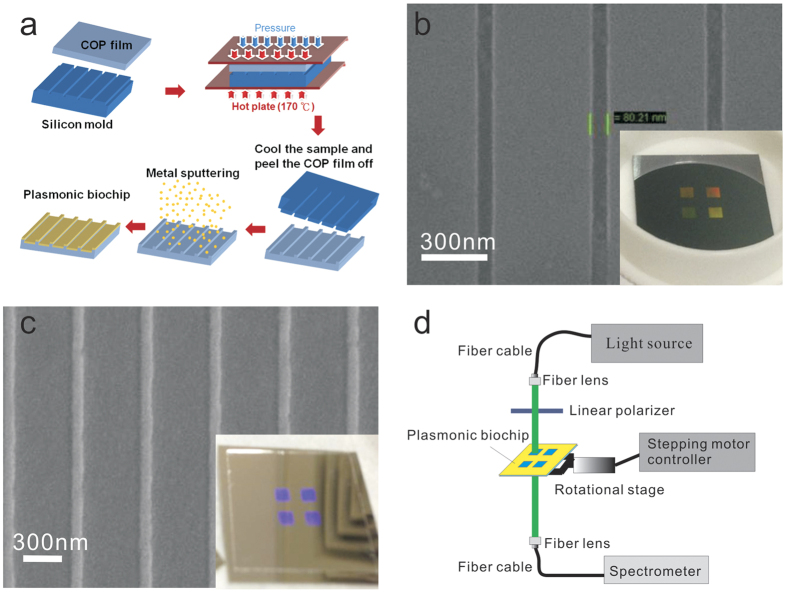
Fabrication of capped nanoslits and optical setup for angular transmission spectrum measurement. (**a**) A process flowchart for the fabrication of silver capped nanoslits. (**b**) The SEM and optical images (inset) of the fabricated silicon template. The groove width is 80 nm and the area of each slit array is 2 mm × 2 mm. (**c**) The SEM and optical images (inset) of the silver capped nanoslits. (**d**) The optical setup for measuring transmission spectra at different angles.

Figure 7a shows a process flowchart for the fabrication of the sliver capped nanoslits. The nanostructures were produced on a cyclic olefin polymer (COP) substrate using hot embossing nanoimprint lithography and metal sputtering. First, periodic nanogrooves of 80 nm in width, 80 nm in depth and 520 nm (or 500 nm) in period were fabricated on a silicon substrate using electron beam lithography and a reactive ion etching method. A 300-nm-thick ZEP-520 resistor (ZEP-520, Zeon Corp, Tokyo, Japan) was spin-coated on a 525-μm-thick silicon substrate. An electron-beam writing system (ELS 7000, Elionix, Japan) was used to write groove arrays with various groove periods. The patterns were then transferred to the silicon substrate using a reactive ion etching machine (Oxford Instrument, plasmalab 80plus). Figure 7b shows the scanning electron microscope (SEM) and optical images (inset) of the fabricated silicon template. The nanostructures on the silicon were then imprinted onto a 178-μm-thick COP film using hot embossing nanoimprint equipment (EHN-3250, Engineering System Co. Ltd.) After sputtering an 80-nm-thick silver film on the imprinted plastic substrate, the silver capped nanoslit arrays were produced. Figure 7c shows the SEM and optical images (inset) of the silver capped nanoslits. There were 4 arrays on the chip. The area of each periodic nanostructure was 2 × 2 mm^2^.

### Optical setup for angular transmission spectrum measurement

Figure 7d shows a simple optical setup for measuring angular transmission spectra. A 150 W white light was coupled to a fiber cable and a fiber lens for light collimation. Its incident polarization was controlled by a linear polarizer. The white light was focused on a capped nanoslit array. To control the incident angle, the sample was put on a rotational stage controlled with a stepper motor. The transmission light was collected by another fiber lens and focused on a fiber cable. The angular transmission spectra were taken using a fiber-coupled linear charge-coupled device (CCD) array spectrometer (BWTEK, BTC112E).

### Refractive index sensitivity tests and biosensing experiments

The bulk refractive index sensitivities were measured by pouring purified water mixed with various fractions of glycerin over the sample surface. The refractive indexes of the mixtures (from 0 to 20% glycerin) ranged from 1.3330 to 1.3575. The biosensing experiments were conducted using bovine serum albumin (BSA, Sigma-Aldrich) and anti-BSA (Sigma-Aldrich) assay in a deionized water buffer. First, 100 μL of 1 mg/mL BSA solution was dropped onto the structure surface for 1 hour. The sample was then washed with ultrapure water to remove the unbound BSA proteins and purged dry by nitrogen gas. Finally, 100 μL of 1 ng/mL anti-BSA solution was dropped onto the structure surface for 1 hour. The sample was then washed with ultrapure water and purged dry by nitrogen gas. The dropping, washing and nitrogen-purged drying processes were subsequently repeated for different concentrations of anti-BSA solutions from 1 ng/mL to 25 μg/mL. The angular transmission spectrum measurements were conducted before and after BSA and anti-BSA adsorption.

## Additional Information

**How to cite this article**: Lee, K.-L. *et al*. Enhancing the Surface Sensitivity of Metallic Nanostructures Using Oblique-Angle-Induced Fano Resonances. *Sci. Rep*. **6**, 33126; doi: 10.1038/srep33126 (2016).

## References

[b1] Raether H. Surface plasmons on smooth and rough surfaces and on gratings (Springer Tracts Mod. Phys. 111, Springer, 1988).

[b2] Homola J., Yee S. S. & Gauglitz G. Surface plasmon resonance sensors: review. Sens. Actuator B-Chem. 54, 3–15 (1999).

[b3] Maier S. A. Plasmonics: fundamentals and applications. (Springer-Verlag, New York, 2007).

[b4] Homola J. Surface plasmon resonance sensors for detection of chemical and biological species. Chem. Rev. 108, 462–493 (2008). 18229953 10.1021/cr068107d

[b5] Anker J. N. . Biosensing with plasmonic nanosensors. Nat. Mater. 7, 442–453 (2008). 18497851 10.1038/nmat2162

[b6] Brolo A. G., Gordon R., Leathem B. & Kavanagh K. L. Surface plasmon sensor based on the enhanced light transmission through arrays of nanoholes in gold films. Langmuir 20, 4813–4815 (2004). 15984236 10.1021/la0493621

[b7] Tetz K. A., Pang L. & Fainman Y. High-resolution surface plasmon resonance sensor based on linewidth-optimized nanohole array transmittance. Opt. Lett. 31, 1528–1530 (2006). 16642161 10.1364/ol.31.001528

[b8] Henzie J., Lee M. H. & Odom T. W. Multiscale patterning of plasmonic metamaterials. Nat. Nanotechnol. 2, 549–554 (2007). 18654366 10.1038/nnano.2007.252

[b9] Yang J. C., Ji J., Hogle J. M. & Larson D. N. Metallic nanohole arrays on fluoropolymer substrates as small label-free real-time bioprobes. Nano Lett. 8, 2718–2724 (2008). 18710296 10.1021/nl801043t PMC2662724

[b10] Lindquist N. C., Lesuffleur A., Im H. & Oh S. H. Sub-micron resolution surface plasmon resonance imaging enabled by nanohole arrays with surrounding Bragg mirrors for enhanced sensitivity and isolation. Lab Chip 9, 382–387 (2009). 19156286 10.1039/b816735d

[b11] Gordon R., Sinton D., Kavanagh K. L. & Brolo A. G. A new generation of sensors based on extraordinary optical transmission. Accounts Chem. Res. 41, 1049–1057 (2008). 10.1021/ar800074d 18605739

[b12] Lee K. L., Lee C. W., Wang W. S. & Wei P. K. Sensitive biosensor array by using surface plasmon resonance on metallic nanoslits. J. Biomed. Opt. 12, 044023 (2007). 17867827 10.1117/1.2772296

[b13] Lee K. L., Huang J. B., Chang J. W., Wu S. H. & Wei P. K. Ultrasensitive biosensors using enhanced Fano resonances in capped gold nanoslit arrays. Sci. Rep. 5, 8547 (2015). 25708955 10.1038/srep08547 PMC4338429

[b14] Pang L., Hwang G. M., Slutsky B. & Fainman Y. Spectral sensitivity of two-dimensional nanohole array surface plasmon polariton resonance sensor. Appl. Phys. Lett. 91, 123112 (2007).

[b15] Lee S. H. . Linewidth-optimized extraordinary optical transmission in water with template-stripped metallic nanohole arrays. Adv. Func. Mat. 22, 4439–4446 (2012).

[b16] Becker J., Trügler A., Jakab A. & Hohenester U. The optimal aspect ratio of gold nanorods for plasmonic bio-sensing. Plasmonics 5, 161–167 (2010).

[b17] Lee K. L., Wu S. H. & Wei P. K. Intensity sensitivity of gold nanostructures and its application for high-throughput biosensing. Opt. Express 17, 23104–23113 (2009). 20052237 10.1364/OE.17.023104

[b18] Kegel L. L., Boyne D. & Booksh K. S. Sensing with prism-based near-infrared surface plasmon resonance spectroscopy on nanohole array platforms. Anal. Chem. 86, 3355–3364 (2014). 24499170 10.1021/ac4035218

[b19] Nagpal P., Lindquist N. C., Oh S. H. & Norris D. J. Ultrasmooth patterned metals for plasmonics and metamaterials. Science 325, 594–597 (2009). 19644116 10.1126/science.1174655

[b20] Hegner M., Wagner P. & Semenza G. Ultralarge atomically flat template-stripped Au surfaces for scanning probe microscopy. Surf. Sci. 291, 39–46 (1993).

[b21] Im H. . Template-stripped smooth Ag nanohole arrays with silica shells for surface plasmon resonance biosensing. ACS Nano 5, 6244–6253 (2011). 21770414 10.1021/nn202013v PMC3160512

[b22] Lee K. L. . Enhancing surface plasmon detection using template-stripped gold nanoslit arrays on plastic films. ACS Nano 6, 2931–2939 (2012). 22452266 10.1021/nn3001142

[b23] Chen K. P., Drachev V. P., Borneman J. D., Kildishev A. V. & Shalaev V. M. Drude relaxation rate in grained gold nanoantennas. Nano Lett. 10, 916–922 (2010). 20128610 10.1021/nl9037246

[b24] Fano U. The theory of anomalous diffraction gratings and of quasi-stationary waves on metallic surfaces (Sommerfeld’s waves). JOSA 31, 213–222 (1941).

[b25] Miroshnichenko A. E., Flach S. & Kivshar Y. S. Fano resonances in nanoscale structures. Rev. Mod. Phys. 82, 2257–2298 (2010).

[b26] Luk’yanchuk B. . The Fano resonance in plasmonic nanostructures and metamaterials. Nat. Mater. 9, 707–715 (2010). 20733610 10.1038/nmat2810

[b27] Yanik A. A. . Seeing protein monolayers with naked eye through plasmonic Fano resonances. Proc. Natl. Acad. Sci. USA 108, 11784–11789 (2011). 21715661 10.1073/pnas.1101910108 PMC3141965

[b28] Liu N. . Planar metamaterial analogue of electromagnetically induced transparency for plasmonic sensing. Nano Lett. 10, 1103–1107 (2010). 20017551 10.1021/nl902621d

[b29] Gao H. . Using the angle-dependent resonances of molded plasmonic crystals to improve the sensitivities of biosensors. Nano Lett. 10, 2549–2554 (2010). 20509678 10.1021/nl101165r

[b30] Tsai W. S., Lee K. L., Pan M. Y. & Wei P. K. Increased detection sensitivity of surface plasmon sensors using oblique induced resonant coupling. Opt. Lett. 38, 4962–4965 (2013). 24281483 10.1364/OL.38.004962

[b31] Lin E. H., Tsai W. S., Lee K. L., Lee M. C. & Wei P. K. Enhancing detection sensitivity of metallic nanostructures by resonant coupling mode and spectral integration analysis. Opt. Express 22, 19621–19632 (2014). 25321045 10.1364/OE.22.019621

[b32] Gordon R. Light in a subwavelength slit in a metal: propagation and reflection. Phys. Rev. B 73, 153405 (2006).

[b33] Lee K. L., Wu S. H., Lee C. W. & Wei P. K. Sensitive biosensors using Fano resonance in single gold nanoslit with periodic grooves. Opt. Express 19, 24530–24539 (2011). 22109480 10.1364/OE.19.024530

[b34] Chang S. H., Gray S. K. & Schatz G. C. Surface plasmon generation and light transmission by isolated nanoholes and arrays of nanoholes in thin metal films. Opt. Express 13, 3150–3165 (2005). 19495214 10.1364/opex.13.003150

[b35] Palik E. D. Handbook of Optical Constant of Solids. (Academic: Florida, 1985).

[b36] Zou S., Janel N. & Schatz. G. C. Silver nanoparticle array structures that produce remarkably narrow plasmon lineshapes. J. Chem. Phys. 120, 10871 (2004). 15268116 10.1063/1.1760740

[b37] Markel V. A. Divergence of dipole sums and the nature of non-Lorentzian exponentially narrow resonances in one-dimensional periodic arrays of nanospheres. J. Phys. B. 38, L115–L121 (2005).

[b38] Kravets V. G., Schedin F. & Grigorenko A. N. Extremely narrow plasmon resonances based on diffraction coupling of localized plasmons in aqrrays of metallic nanoparticles. Phys. Rev. Lett. 101, 087403 (2008). 18764660 10.1103/PhysRevLett.101.087403

[b39] Auguié B. & Barnes W. L. Collective resonances in gold nanoparticle arrays. Phys. Rev. Lett. 101, 143902 (2008). 18851529 10.1103/PhysRevLett.101.143902

[b40] Martin-Moreno L. . Theory of extraordinary optical transmission through subwavelength hole arrays. Phys. ReV. Lett. 86, 1114–1117 (2001). 11178023 10.1103/PhysRevLett.86.1114

[b41] Krishnan A. . Evanescently coupled resonance in surface plasmon enhanced transmission. Opt. Commun. 200, 1–7 (2001).

[b42] Yang J. C., Ji J., Hogle J. M. & Larson D. N. Metallic nanohole arrays on fluoropolymer substrates as small label-free real-time bioprobes. Nano Lett. 8, 2718–2724 (2008). 18710296 10.1021/nl801043t PMC2662724

[b43] Ying P., Yu Y., Jin G. & Tao Z., Competitive protein adsorption studied with atomic force microscopy and imaging ellipsometry. Colloids Surf. B Biointerfaces 32, 1–10 (2003).

[b44] Pan M. Y., Lee K. L., Tsai W. S., Wang L. & Wei P. K. Determination of the effective index and thickness of biomolecular layer by Fano resonances in gold nanogrid array. Opt. Express 23, 21596–21606 (2015). 26368138 10.1364/OE.23.021596

[b45] Stenberg E., Persson B., Roos H. & Urbaniczky C. Quantitative determination of surface concentration of proteins with surface plasmon resonance using radiolabeled protein. J. Colloid. Interf. Sci. 143, 513–526 (1991).

[b46] Schasfoort M. & Tudos A. J. (Eds) Handbook of Surface Plasmon Resonance. (Royal Society of Chemistry, 2008).

[b47] Liedberg B., Nylander C. & Lundström I. Biosensing with surface plasmon resonance how it all started. Biosens. Bioelectron 10, i–ix (1995). 7576432 10.1016/0956-5663(95)96965-2

[b48] Kravets V. G. . Singular phase nano-optics in plasmonic metamaterials for label-free single-molecule detection. Nature Mater. 12, 304–309 (2013). 23314104 10.1038/nmat3537

[b49] Kravets V. G. . Graphene-protected copper and silver plasmonics. Sci. Rep. 4, 5517 (2014). 24980150 10.1038/srep05517 PMC4076691

